# Revisiting random walk based sampling in networks: evasion of burn-in period and frequent regenerations

**DOI:** 10.1186/s40649-018-0051-0

**Published:** 2018-03-19

**Authors:** Konstantin Avrachenkov, Vivek S. Borkar, Arun Kadavankandy, Jithin K. Sreedharan

**Affiliations:** 1 INRIA Sophia Antipolis, Valbonne, France; 2 IIT Bombay, Mumbai, India; 3 CentraleSupélec, Gif-sur-Yvette, France; 4 Purdue University, West Lafayette, USA

**Keywords:** Network sampling, Random walks on graph, Reinforcement learning, Respondent-driven sampling

## Abstract

**Background:**

In the framework of network sampling, random walk (RW) based estimation techniques provide many pragmatic solutions while uncovering the unknown network as little as possible. Despite several theoretical advances in this area, RW based sampling techniques usually make a strong assumption that the samples are in stationary regime, and hence are impelled to leave out the samples collected during the burn-in period.

**Methods:**

This work proposes two sampling schemes without burn-in time constraint to estimate the average of an arbitrary function defined on the network nodes, for example, the average age of users in a social network. The central idea of the algorithms lies in exploiting regeneration of RWs at revisits to an aggregated super-node or to a set of nodes, and in strategies to enhance the frequency of such regenerations either by contracting the graph or by making the hitting set larger. Our first algorithm, which is based on reinforcement learning (RL), uses stochastic approximation to derive an estimator. This method can be seen as intermediate between purely stochastic Markov chain Monte Carlo iterations and deterministic relative value iterations. The second algorithm, which we call the Ratio with Tours (RT)-estimator, is a modified form of respondent-driven sampling (RDS) that accommodates the idea of regeneration.

**Results:**

We study the methods via simulations on real networks. We observe that the trajectories of RL-estimator are much more stable than those of standard random walk based estimation procedures, and its error performance is comparable to that of respondent-driven sampling (RDS) which has a smaller asymptotic variance than many other estimators. Simulation studies also show that the mean squared error of RT-estimator decays much faster than that of RDS with time.

**Conclusion:**

The newly developed RW based estimators (RL- and RT-estimators) allow to avoid burn-in period, provide better control of stability along the sample path, and overall reduce the estimation time. Our estimators can be applied in social and complex networks.

## Background

The prohibitive sizes of most social networks make graph-processing that requires complete knowledge of the graph impractical. For instance, social networks like Facebook or Twitter have billions of edges and nodes. In such a situation, we address the problem of estimating global properties of a large network to some degree of accuracy. Some examples of potentially interesting properties include the size of the support base of a certain political party, the average age of users in an online social network (OSN), the proportion of male–female connections with respect to the number of female–female connections in an OSN, and many others. Naturally, since graphs can be used to represent data in myriad of disciplines and scenarios, the questions we can ask are endless.

Graph sampling is a possible solution to address the above problem. To collect information from an OSN, the sampler issues an Application Programming Interface (API) query for a particular user, which returns its one-hop neighborhood and the contents published. Though some OSNs (for instance Twitter) allow access to the complete database with additional expense, we focus here on the typical case when a sampler can get information only about the neighbors of a particular user by means of API queries. There are several ways to collect representative samples in a network. One straightforward way is to collect independent samples via uniform node or edge sampling. However, uniform sampling is not efficient because we do not know the user ID space beforehand. Consequently, the sampler wastes many samples issuing invalid IDs resulting in an inefficient and costly data collection method. Moreover, OSNs typically impose rate limitations on the API queries, e.g., Twitter with 313 million active users enforces a limit of 15 on requests in a 15-min time window for most of APIs.^1^ With this limitation, the crawler will need 610 years to crawl the whole Twitter. Therefore, if only a standard API is available to us, we inevitably need to use some sampling technique, and RW based techniques appear as a good option.

### Important notation and problem formulation

Let $$G=(V,E)$$ be an undirected labeled network, where *V* is the set of vertices and $$E \subseteq V \times V$$ is the set of edges. Although the graph is undirected, in later use it would be more convenient to represent edges by ordered pairs (*u*, *v*). Of course, if $$(u,v) \in E,$$ it holds that $$(v,u) \in E,$$ since *G* is undirected. With a slight abuse of notation, the total number of undirected edges $$|E |/2 $$ is denoted as $${|E |}.$$

Both edges and nodes can have function values defined on them. For instance, in an OSN, the node function can be the age or number of friends and the edge function can be an indicator function when the terminal nodes of the edge are of same gender. Let us denote by $g: V \rightarrow \mathbb R,$ where $\mathbb R$ is the real number space, a function on the vertices of the graph. We aim to estimate the following network function average:

1 $$\begin{aligned} \nu (G) = \frac{1}{{{ |V |}}} \sum _{u \in V} g(u). \end{aligned}$$

The constraint on the estimator is that it does not know the whole graph, and can only issue API requests. Each API request furnishes the function value $g(\cdot )$ at the node queried and the list of its neighbors. Let $\hat{\nu }^{(n)}_{\text {XY}}(G)$ be our estimate of $\nu (G)$ formed from *n* samples using the scheme XY. We will occasionally drop *n* and the scheme if it is clear from the context.

A *simple RW* (SRW) on a graph offers a viable solution to this problem that respects the above constraints. From an initial node, a simple RW proceeds by choosing one of the neighbors uniformly randomly and repeating the same process at the next node and so on. In general, a RW need not sample the neighbors uniformly and can take any transition probability compliant with the underlying graph, an example being the Metropolis–Hastings schemes [1]. Random walk techniques are well known (see for instance [2–11] and references therein). A drawback of random walk techniques is that they all suffer from the problem of initial burn-in, i.e., a number of initial samples need to be discarded to get samples from a desired probability distribution. The burn-in period (or mixing time) of a random walk is the time period after which the RW produces almost stationary samples irrespective of the initial distribution. This poses serious limitations, especially in view of the stringent constraints on the number of samples imposed by API query rates. In addition, subsequent samples of a RW are obviously not independent. To get independent samples, it is customary to drop intermediate samples. *In this work, we focus on RW based algorithms that bypass this burn-in time barrier*. We focus on two approaches: reinforcement learning and tour-based ratio estimator.

### Related work and contributions

Many random walk based sampling techniques have been introduced and studied in detail recently. The estimation techniques in [2–5] avoid the burn-in time drawback of random walks, similar to our aim. The works [2–4] are based on the idea of a random walk tour, which is a sample path of a random walk starting and ending at a fixed node. Massoulié et al. [3] estimate the size of a network based on the return times of RW tours. Cooper et al. [2] estimate the number of triangles, network size, and subgraph counts from weighted random walk tours using the results of Aldous and Fill [12, Chapters 2 and 3]. The work in [4] extends these results to edge functions, provides real-time Bayesian guarantees for the performance of the estimator, and introduced some hypothesis tests using the estimator. Instead of the estimators for the sum function of the form $\sum _{u \in V} g(u)$ proposed in these previous works; here we study the average function (1). Walk-estimate proposed in [5] aimed to reduce the overhead of burn-in period by considering short random walks and then using acceptance–rejection sampling to adjust the sampling probability of a node with respect to its stationary distribution. This work requires an estimate of probability of hitting a node at time *t*, which introduces a computational overhead. It also needs an estimate of the graph diameter to work correctly. Our algorithms are completely local and do not require these global inputs.

There are also specific random walk methods tailored for certain forms of function *g*(*v*) or criterion, for instance, in [10] the authors developed an efficient estimation technique for estimating the average degree, and Frontier sampling in [11] introduced dependent multiple random walks in order to reduce estimation error.

Two well-known techniques for estimating network averages $\nu (G)$ are the Metropolis–Hastings MCMC (MH-MCMC) scheme [1, 9, 13, 14] and Respondent-Driven sampling (RDS) [6–8].

In our work, we first present a theoretical comparison of the mean-squared error of MH-MCMC and RDS estimators. It was observed that in many practical cases RDS outperforms MH-MCMC in terms of asymptotic error. We confirm this observation here using theoretical expressions for the asymptotic mean-squared errors of the two estimators. Then, we introduce a novel estimator for the network average based on reinforcement learning (RL). By way of simulations on real networks, we demonstrate that, with a good choice of cooling schedule, RL can achieve similar asymptotic error performance to RDS but its trajectories have smaller fluctuations.

Finally, we extend RDS to accommodate the idea of regeneration during revisits to a node or to a ‘super-node,’ formed by aggregating several nodes, and propose the RT-estimator, which does not suffer from burn-in period constraints and significantly outperforms the RDS estimator.

### Notational conventions

Expectation w.r.t. the initial distribution $\eta$ of a RW is denoted by $\mathbb E_{\eta }$, and if the distribution degenerates at a particular node *j*, the expectation is $\mathbb {E} _j$. Matrices in $\mathbb R^{n \times n}$ are denoted by boldface uppercase letters, e.g., $\varvec{A}$, and vectors in $\mathbb R^{n \times 1}$ are denoted by lowercase boldface letters, e.g., $\varvec{x}$, whereas their respective elements are denoted by non-bold letters, e.g., $A_{ij},x_i.$ Convergence in distribution is denoted by $\xrightarrow {D}.$ By $\mathcal L(X)$ we mean the law or the probability distribution of a random variable. We use $\mathcal {N}(\mu ,\sigma ^2)$ to denote a Gaussian random variable with mean $\mu$ and variance $\sigma ^2.$ Let us define the fundamental matrix of a Markov chain as $\varvec{Z} := (\varvec{I} - \varvec{P} + \mathbf{1}{\mathbf {\pi }}^{\intercal })^{-1}.$ For two functions $f,g: \mathcal {V}\rightarrow \mathbb {R},$ we define $\sigma ^2_{ff} := 2 \langle \varvec{f}, \varvec{Z} \varvec{f} \rangle _{\pi } - \langle f,f \rangle _{\pi } - \langle f,\mathbf{1}{\mathbf {\pi }}^{\intercal }f \rangle _{\pi },$ and $\sigma ^2_{fg} := \langle \varvec{f}, \varvec{Z} \varvec{g} \rangle _{\pi } + \langle \varvec{g}, \varvec{Z} \varvec{f} \rangle _{\pi } - \langle f,g \rangle _{\pi } - \langle f,\mathbf{1}{\mathbf {\pi }}^{\intercal } g \rangle _{\pi },$ where $\langle \varvec{x},\varvec{y} \rangle _{\pi } := \sum _{i} x_i y_i \pi _i,$ for any two vectors $\varvec{x},\varvec{y} \in \mathbb {R}^{|\mathcal {V}| \times 1}, \pi$ being the stationary distribution of the Markov chain.

### Organization

The rest of the paper is organized as follows: In “MH-MCMC and RDS estimators” section, we discuss MH-MCMC as well as RDS providing both known and new material on the asymptotic variance of the estimators.  “Network sampling with reinforcement learning (RL-technique)” section introduces the reinforcement learning based technique for sampling and estimation. It also details a modification for an easy implementation with SRW. Then, in  “Ratio with tours estimator (RT-estimator)” section, we introduce a modification of the RDS based on the ideas of super-nodes and tours. This modification also does not have a burn-in period and significantly outperforms the plain RDS.  “Numerical results” section contains numerical simulations performed on real-world networks and makes several observations about the new algorithms. We conclude the article with “Conclusions” section.

## MH-MCMC and RDS estimators

The utility of RW based methods comes from the fact that for any initial distribution $\nu,$ as time progresses, the sample distribution of the RW at time *t* starts to resemble a fixed distribution, which we call the stationary distribution of the RW, denoted by $\pi .$

We will study mean squared error and asymptotic variance of random walk based estimators in this paper. For this purpose, following extension of the central limit theorem for Markov chains plays a significant role:

### Theorem 1

([15]) *Let f be a real-valued function* $f: V \mapsto \mathbb {R}$* with* $\mathbb {E} _{\pi }[f^2(X_0)] < \infty$*. For a finite irreducible Markov chain* $\{X_n\}$* with stationary distribution* $\pi,$

$$\begin{aligned} \sqrt{n} \left( \frac{1}{n} \sum _{k=0}^{n-1} f(X_k) - \mathbb {E} _{\pi }[f(X_0)] \right) \xrightarrow {D} \mathcal {N}(0,\sigma ^2_f), \end{aligned}$$

*irrespective of the initial distribution, where*

2 $$\begin{aligned} \sigma ^2_f&= \lim _{n \rightarrow \infty } n \times \mathbb {E} \left[ {\left\{ \frac{1}{n} \sum _{k=0}^{n-1} f(X_k) - \mathbb {E} _{\pi }[f(X_0)] \right\} }^2 \right] \nonumber \\&: = \lim _{n \rightarrow \infty } \frac{1}{n} {\mathrm{Var}} \left[ \sum _{k=0}^{n-1} f(X_k) \right] . \end{aligned}$$

Note that, the above also holds for finite periodic chains (with the existence of unique solution to ${\mathbf {\pi }}^{\intercal } \varvec{P} = {\mathbf {\pi }}^{\intercal }$).

By [13, Theorem 6.5] $\sigma ^2_f$ in Theorem 1 is the same as $\sigma ^2_{ff}.$ We will also need the following theorem.

### Theorem 2

([14], Theorem 3)* If f, g are two functions defined on the states of a random walk, define the vector sequence* $$\mathbf {Z}_k = \left[ \begin{matrix} f(X_k) \\ g(X_k) \end{matrix} \right] $$ *the following central limit theorem holds*

$$\begin{aligned} \sqrt{n} \left( \frac{1}{n} \sum _{k = 1}^n \mathbf {Z}_k - \mathbb {E} _{\pi }(\mathbf {Z}_k)\right) \xrightarrow {D} \mathcal {N}(0,\mathbf {\Sigma }), \end{aligned}$$

*where* $\mathbf {\Sigma }$* is* $2 \times 2$ *matrix such that* $\mathbf {\Sigma }_{11} = \sigma ^2_{ff},\mathbf {\Sigma }_{22} = \sigma ^2_{gg}$* and* $\mathbf {\Sigma }_{12} = \mathbf {\Sigma }_{21}= \sigma ^2_{fg}.$

The time required by a random walk or Markov chain to reach stationarity is measured by a parameter called *mixing time* defined as

$$\begin{aligned} t_{\mathrm{mix}}(\epsilon ) := \min \{t: \max _{u \in V} {\Vert {\varvec{P}}^t(x,\cdot ) - \mathbf {\pi } \Vert }_{\mathrm{TV}} \le \epsilon \}, \end{aligned}$$

where ${\Vert \xi _1 - \xi _2 \Vert }_{\text {TV}} : = \max _{A \subset V} |\xi _1(A) - \xi _2(A) |$ is the total variational distance between the probability distributions $\xi _1$ and $\xi _2$. If the mixing time is known, then as many samples are omitted in any RW based algorithm to ensure that the samples are in stationary regime. Since it is difficult to calculate the mixing time accurately, practitioners often use a prediction called burn-in period which is much larger than the mixing time.

### Function average from RWs

The SRW is biased towards higher degree nodes and by Theorem 1, the sample averages converge to the stationary average. Hence if the aim is to estimate an average function (1), the RW needs to have uniform stationary distribution. Alternatively, the RW should be able to unbias it locally. In order to obtain the average, we modify the function *g* by normalizing it by the vertex degrees to get $g'(u) = g(u)/\mathbf {\pi }_{u},$ where $\pi _u = d_u/(2|E|).$ Since $\mathbf {\pi }(u)$ contains ${ |E |}$ and the knowledge of ${ |E |}$ is not available to us initially, it also needs to be estimated. To overcome this problem, we consider the following modifications of the SRW-based estimator.

### Metropolis–Hastings random walk

We review here the Metropolis–Hastings MCMC (MH-MCMC) algorithm. When the chain is in state *i*, it chooses the next state *j* according to transition probability $p_{ij}$. It then jumps to this state with probability $q_{ij}$ or remains in the current state *i* with probability $1 - q_{ij},$ where $q_{ij}$ is given as below:

3 $$\begin{aligned} q_{ij} = {\left\{ \begin{array}{ll} \min \left( \frac{ p_{ji} }{ p_{ij} } , 1 \right) &{} \text { if } p_{ij} > 0, \\ 1 &{} \text { if } p_{ij} = 0. \end{array}\right. } \end{aligned}$$

Therefore, the effective jump probability from state *i* to state *j* is $q_{ij}p_{ij},$ when $i \ne j.$ It follows then that such a process represents a Markov chain with the following transition matrix ${\varvec{P}}^{\text {MH}}$

$$\begin{aligned} {{P}}^{\text {MH}}_{ij} = {\left\{ \begin{array}{ll} \frac{1}{\max ({d_i,d_j})} &{} \text { if } (i,j) \in E, \\ 1 - \sum _{k \ne i} \frac{1}{\max ({d_i,d_k})} &{} \text { if } i =j, \\ 0 &{} \text { if } (i,j) \notin E, i \ne j. \end{array}\right. } \end{aligned}$$

This chain is reversible with stationary distribution $\varvec{\pi }(i) = 1/n \ \forall i \in V$. Therefore, the following estimate for $\nu (G)$ using MH-MCMC, $\{X_n\}$ being MH-MCMC samples, is asymptotically consistent.

$$\begin{aligned} \widehat{\nu }^{(n)}_{\text {MH}}(G) = \frac{1}{n} \sum _{k=1}^n g(X_k) \end{aligned}$$

By using Theorem 1, we can show the following central limit theorem for MH-MCMC.

#### Proposition 1

(Central Limit Theorem for MH-MCMC)* For MCMC with uniform target stationary distribution it holds that*

$$\begin{aligned} \sqrt{n} \left( \widehat{\nu }^{(n)}_{\mathrm{MH}}(G) - \nu (G)\right) \xrightarrow {D} \mathcal {N}(0,\sigma ^2_{\mathrm{MH}}), \end{aligned}$$

*as* $n \rightarrow \infty ,$* where* $\sigma ^2_{\mathrm{MH}} = \sigma ^2_{gg} =\frac{2}{|V|} {\mathbf {g}}^{\intercal } \varvec{Z}^{\mathrm{MH}} \mathbf {g} - \frac{1}{|V|} {\mathbf {g}}^{\intercal } \mathbf {g} - \left( \frac{1}{|V|} {\mathbf {g}}^{\intercal } \mathbf{1}\right) ^2$ *and* $\varvec{Z}^{\mathrm{MH}} = (\varvec{I} - \varvec{P}^{\mathrm{MH}} + \frac{1}{|V|}\mathbf{1}\mathbf{1}^{\intercal })^{-1}.$

### Respondent-driven sampling technique (RDS-technique)

The estimator with respondent-driven sampling uses the SRW on graphs but applies a correction to the estimator to compensate for the non-uniform stationary distribution, i.e.,

4 $$\begin{aligned} \widehat{\nu }^{(n)}_{\text {RDS}}(G) = \frac{\sum _{k=1}^n g(X_k)/d(X_k)}{\sum _{k=1}^n 1/d(X_k)}. \end{aligned}$$

We define $h_{\text {nm}}(X_k) : = g(X_k)/d(X_k),h_{\text {dm}}(X_k) : = 1/d(X_k)$. Note that this estimator does not require |*E*|.

The asymptotic unbiasedness derives from the Ergodic Theorem and also as a consequence of the CLT given below.

Now we have the following CLT for the RDS Estimator.

#### Proposition 2

*The RDS estimator* $\widehat{\nu }^{(n)}_{\mathrm{RDS}}(G)$ *satisfies a central limit theorem given below*

$$\begin{aligned} \sqrt{n} \left( \widehat{\nu }^{(n)}_{\mathrm{RDS}}(G) - \nu (G) \right) \xrightarrow {D} {\mathrm{Normal}}(0,\sigma ^2_{\mathrm{RDS}}), \end{aligned}$$

*where* $\sigma ^2_{\mathrm{RDS}}$ *is given by*

$$\begin{aligned} \sigma ^2_{\mathrm{RDS}} = d_{\mathrm{av}}^2 \left( \sigma _1^2 + \sigma _2^2 \nu ^2(G) - 2 \nu (G) \sigma _{12}^2 \right) , \end{aligned}$$

*where* $$\sigma _1^2 = \frac{1}{|E|}\sum _{i,j \in V} g_i Z_{ij}g_j/d_j - \frac{1}{2|E|}\sum _{i\in V} \frac{g_i}{d_i} - (\frac{1}{2|E|} \sum _{i \in V}g_i)^2, \sigma _2^2 = \frac{1}{|E|} \sum _{i,j \in V} Z_{ij}/d_j - \frac{1}{2|E|}\sum _i \frac{1}{d_i} - (\frac{1}{d_{\mathrm{av}}})^2 ,\sigma _{12}^2 = \frac{1}{2|E|}\sum _{i,j\in V}g_i Z_{ij}/d_j + \frac{1}{2|E|} \sum _{i,j \in V} Z_{ij}/d_i - \frac{1}{2|E|d_{av} }\sum _i g_i. $$

#### *Proof*

Define the vector $$\varvec{z}_t = \left[ \begin{matrix} h_{\text {nm}}(x_t) \\ h_{\text {dm}}(x_t) \end{matrix} \right] ,$$ and let $\widetilde{\varvec{z}}_n = \sqrt{n} \left( \frac{1}{n} \sum _{t = 1}^n \varvec{z}_t - \mathbb {E}_{\pi }(\varvec{z}_t)\right) .$ Then by Theorem 2, $\widetilde{\varvec{z}}_n \xrightarrow {\mathcal {D}} \mathrm{Normal}(0,\varvec{\Sigma }),$ where $\varvec{\Sigma }$ is the correlation matrix, whose formula given in Theorem 2. Let $\widetilde{\varvec{z}}_n = (\widetilde{\varvec{z}}^1_n,\widetilde{\varvec{z}}^2_n).$ Then we have

$$\begin{aligned} {\frac{\sum _{t=1}^n h_{\text {nm}}(x_t)}{\sum _{t=1}^n h_{\text {dm}}(x_t)}}&= \frac{\frac{1}{\sqrt{n}} \widetilde{\varvec{z}}^1_n + \mu _{h_{\text {nm}}}}{\frac{1}{\sqrt{n}} \widetilde{\varvec{z}}^2_n + \mu _{h_{\text {dm}}}}\\&= \frac{\widetilde{\varvec{z}}^1_n + \sqrt{n} \mu _{h_{\text {nm}}} }{\widetilde{\varvec{z}}^2_n + \sqrt{n} \mu _{h_{\text {dm}}}} = \frac{ \widetilde{\varvec{z}}^1_n + \sqrt{n} \mu _{h_{\text {nm}}}}{\sqrt{n} \mu _{h_{\text {dm}}} \left(1 + \frac{\widetilde{\varvec{z}}^2_n}{\sqrt{n} \mu _{h_{\text {dm}}}}\right)} \\&= \frac{1}{\sqrt{n} \mu _{h_{\text {dm}}}} \left(\widetilde{\varvec{z}}^1_n - \frac{\widetilde{\varvec{z}}^1_n\widetilde{\varvec{z}}^2_n}{\sqrt{n} \mu _{h_{\text {dm}}}} + \sqrt{n} \mu _{h_{\text {nm}}} - \frac{\widetilde{\varvec{z}}^2_n \mu _{h_{\text {nm}}}}{\mu _{h_{\text {dm}}}} + \mathcal {O}_p\left(\frac{1}{\sqrt{n}}\right)\right), \end{aligned}$$

where $\mathcal O_p(\frac{1}{\sqrt{n}})$ is a term that goes to zero in probability at least as fast as $\frac{1}{\sqrt{n}},$ and $\mu _{h_{\text {nm}}},\mu _{h_{\text {dm}}}$ are, respectively, $\mathbb E_{\pi }(h_{\text {nm}})$ and $\mathbb E_{\pi }(h_{\text {dm}}).$ Then

5 $$\begin{aligned} {\lim _{n\rightarrow \infty } \mathcal L \left( \sqrt{n} \left( \frac{\sum _{t=1}^n f^{'}(X_t)}{\sum _{t=1}^n g(X_t)} - \frac{\mu _{f^{'}}}{\mu _g}\right) \right) }= & {} \lim _{n\rightarrow \infty } \mathcal L \left( \frac{1}{\mu _g} \left( \widetilde{\varvec{z}}^1_n - \widetilde{\varvec{z}}^2_n \frac{\mu _{f^{'}}}{\mu _{g}}\right) \right) , \end{aligned}$$

by Slutsky’s lemma [16]. The result then follows since $(\widetilde{\varvec{z}}^1_n,\widetilde{\varvec{z}}^2_n)$ converges to jointly Gaussian random variable, and by continuous mapping theorem. $\square$

### Comparing random walk techniques

Random walks can be compared in many ways. Two prominent ways to compare RW estimators are based on their mixing times $t_{\mathrm{mix}}$ and their asymptotic variances. Mixing time is relevant in the situations where the speed at which the RW approaches the stationary distribution matters. But many MCMC algorithms discard some initial samples (called burn-in period) to mitigate the dependence on the initial distribution and this amounts to the mixing time. After the burn-in period, the number of samples needed for achieving a certain estimation accuracy can be determined from the Gaussian approximation given by the central limit theorem (see Theorem 1). Hence, another measure for comparison of the random walks is the asymptotic variance in the Gaussian approximation. The lower the asymptotic variance, the smaller the number of samples needed for a certain estimation accuracy. Many authors consider asymptotic unbiasedness as the principal parameter to compare RW based estimators. For instance, the authors in [17] prove that non-backtracking random walks perform better than the SRW and MH-MCMC methods in terms of the asymptotic variance of the estimators. The asymptotic variance can be related to the eigenvalues of $\varvec{P}$ as follows:

$$\begin{aligned}\sigma^2_f = \sum_{i=2}^{{|V|}} \frac{1+\lambda_i^{\varvec{P}}}{1 - \lambda_i^{\varvec{P}}} \,{\lvert {\langle f, \varvec{u}_i^{\scriptscriptstyle {\varvec{P}}} \rangle}_{\pi} \rvert}^2,\end{aligned}$$

where ${\langle \mathbf {x}, \mathbf {y} \rangle }_{\pi } = \sum _{i \in V} \mathbf {x}_i \mathbf {y}_i \mathbf {\pi }_i$ [13, Chapter 6]. When the interest is in the speed of convergence to equilibrium, then only the second-largest eigenvalue modulus matters. However, if the aim is to compute $\mathbb {E} _{\pi }[ f(X_0) ]$ as the ergodic mean $\lim _{n \rightarrow \infty } \frac{1}{n} \sum _{k=1}^n f(X_k)$, then all the eigenvalues become significant and this is captured when the quality of the ergodic estimator is measured by the asymptotic variance.

## Network sampling with reinforcement learning (RL-technique)

We will now introduce a reinforcement learning approach based on stochastic approximation to estimate $\nu (G)$. The underlying idea relies on the idea of tours and regeneration introduced in [2–4]. We will compare the mean squared error of the new estimator with that of MH-MCMC and RDS, and see how the stability of the sample paths can be controlled.

### Estimator

Let $V_0 \subset V$ with $|V_0|<< |V|$. We assume that the nodes inside $V_0$ are known beforehand. Consider a simple random walk $\{X_n\}$ on *G* with transition probabilities $p(j|i) = 1/d(i)$ if $(i,j) \in E$ and zero otherwise. A random walk tour is defined as the sequence of nodes visited by the random walk during successive return to the set $V_0$. Let $\tau _n :=$ successive times to visit $V_0$ and let $\xi _k : = \tau _k-\tau _{k-1}$. We denote the nodes visited in the *k*th tour as $X_1^{(k)}, X_2^{(k)},\ldots , X_{\xi _k}^{(k)}$. Note that considering $V_0$ helps to tackle a disconnected graph^2^ with RW theory and makes tours shorter. Moreover, the tours are independent of each other and can have *massively parallel implementation*. The estimators derived below and later in “Ratio with tours estimator (RT-estimator)” section exploit the independence of the tours and the result that expected sum of functions of nodes visited in a tour is proportional to $\sum _{u \in V} g(u)$ [4, Lemma 3].

Define $Y_n := X_{\tau _n}$. Then $\{(Y_n, \tau _n)\}$ is a semi-Markov process on $V_0$ [18, Chapter 5]. In particular, $\{Y_n\}$ is a Markov chain on $V_0$ with transition probability matrix (say) $[p_Y(j|i)]$. We have $\xi _1 := \min \{n > 0: X_n \in V_0\}$. For a prescribed $g: V \mapsto \mathbb {R}$, define

$$\begin{aligned} T_i&: =\mathbb {E} _i[\xi _1], \\ h(i)&: =\mathbb {E} _i\left[ \sum _{m=1}^{\xi _1}g(X_m)\right] , \ i \in V_0. \end{aligned}$$

Consider an average cost Markov decision problem (MDP), then the Poisson equation for the semi-Markov process $\{(Y_n, \tau _n)\}$ is [18, Chapter 7]

6 $$\begin{aligned} \mathcal {V}(i) = h(i) - \beta T_i + \sum _{j \in V_0}p_Y(j|i)\mathcal {V}(j), \ i \in V_0, \end{aligned}$$

which is to be solved for the pair $(\mathcal {V},\beta )$, where $\mathcal {V}: V_0 \mapsto \mathbb {R}$ and $\beta \in \mathbb {R}$. Under mild conditions, (6) has the solution $(V^*, \beta ^*)$. The optimal $\beta ^*$ is the average expected cost stationary average of *g*, $\mathbb {E} _{\pi }[g(X_1)]$ [18, Theorem 7.6]. In the following, we provide numerical ways to solve (6). This could be achieved using the classical MDP methods like relative value iteration; instead we look for solutions from reinforcement learning in which the knowledge of transition probability $[p_Y(j|i)]$ is not needed. Stochastic approximation provides a simple and easily tunable solution as follows. The relative value iteration algorithm to solve (6) is

7 $$\begin{aligned} \mathcal {V}_{n+1}(i) = h(i) - \mathcal {V}_n(i_0) T_i + \sum _j p_Y(j|i)V_n(j). \end{aligned}$$

We can implement this using stochastic approximation as follows: let $\{Z_n, n \ge 1 \}$ be from the stationary distribution of the underlying RW conditioned on being in $V_0$. Construct a tour for $n\ge 1$ by starting a SRW $X^{(n)}_i, i \ge 0,$ with $X^{(n)}_0 = Z_n$ and observing its sample path until it returns to $V_0$.

A learning algorithm for (6) along the lines of [19] then is, for $i \in V_0$,

8 $$\begin{aligned} \mathcal {V}_{n+1}(i) & =  {} \mathcal {V}_n(i) \ + a(n)\mathbb {I}\{Z_n = i\} \times \nonumber \\&\quad \left[ \left( \sum _{m=1}^{\xi _n}g(X^{(n)}_m)\right) - \mathcal {V}_n(i_0)\xi _n + \mathcal {V}_n(X^{(n)}_{\xi _n}) - \mathcal {V}_n(i)\right] , \end{aligned}$$

where $a(n) > 0$ are stepsizes satisfying $\sum _n a(n) = \infty , \ \sum _n a(n)^2 < \infty$. (One good choice is $a(n) = 1/\lceil \frac{n}{N}\rceil$ for $N = 50$ or 100.) Here $\mathbb {I}\{A\}$ denotes indicator function for the set *A*. Also, $i_0$ is a prescribed element of $V_0$. One can use other normalizations in place of $\mathcal {V}_n(i_0)$, such as $\frac{1}{|V_0|}\sum _j \mathcal {V}_n(j)$ or $\min _i \mathcal {V}_n(i)$, see e.g., [20]. Then this normalizing term ($\mathcal {V}_n(i_0)$ in (8)) converges to $\beta ^*$, $\mathbb {E} _{\pi }[g(X_1)]$, as *n* increases to $\infty .$

Taking expectations on both sides of (8), we obtain a deterministic iteration that can be viewed as an incremental version of the relative value iteration (7) with suitably scaled stepsize $\tilde{a}(n) := \frac{a(n)}{|V|}$. This can be analyzed the same way as the stochastic approximation scheme with the same o.d.e. limit and therefore the same (deterministic) asymptotic limit. This establishes the asymptotic unbiasedness of the RL estimator.

The normalizing term used in (8) ($\mathcal {V}_n(i_0)$, $\frac{1}{|V_0|}\sum _j \mathcal {V}_n(j)$ or $\min _i \mathcal {V}_n(i)$), along with the underlying random walk as the Metropolis–Hastings, forms our estimator $\widehat{\nu }_{\text {RL}}(G)$ in RL-based approach. The iteration in (8) is the stochastic approximation analog of it which replaces conditional expectation w.r.t. transition probabilities with an actual sample and then makes an incremental correction based on it, with a slowly decreasing stepsize that ensures averaging. The latter is a standard aspect of stochastic approximation theory. The smaller the stepsize, the less the fluctuations but slower the speed; thus, there is a trade-off between the two.

RL methods can be thought of as a cross between a pure deterministic iteration such as the relative value iteration above and pure MCMC, trading off variance against per iterate computation. The gain is significant if the number of neighbors of a node is much smaller than the number of nodes, because we are essentially replacing averaging over the latter by averaging over neighbors. The $\mathcal {V}$-dependent terms can be thought of as control variates to reduce variance.

### Extension of RL-technique to uniform stationary average case

The stochastic approximation iteration in (8) converges to $\beta$, which is $\mathbb {E} _\pi [g(X_1)],$ where $\pi$ is the stationary distribution of the underlying walk. To make it converge to ${\nu }(G)$, we can use the Metropolis–Hastings random walk with uniform target distribution. However, we can avoid the use of Metropolis–Hastings algorithm by the following modification, motivated from importance sampling that achieves the convergence to ${\nu }(G)$ with the simple random walk (SRW). We propose

$$\begin{aligned} {\mathcal {V}_{n+1}(i)}&= \mathcal {V}_n(i) \ + a(n)\mathbb {I}\{z = i\} \times \Gamma _{\xi _n}^{(n)} \times \\&\quad \left[ \left( \sum _{m=1}^{\xi _n}g(X^{(n)}_m)\right) - \mathcal {V}_n(i_0)\xi _n + \mathcal {V}_n(X^{(n)}_{\xi _n}) - \mathcal {V}_n(i)\right] , \end{aligned}$$

where

$$\begin{aligned} \Gamma _{m}^{(n)} = \prod _{k=1}^m \left( \frac{p(X_k^{(n)} | X_{k-1}^{(n)})}{q(X_k^{(n)} | X_{k-1}^{(n)})} \right) . \end{aligned}$$

Here $q(\cdot |\cdot )$ is the transition probability of the random walk with which we simulate the algorithm and $p(\cdot |\cdot )$ corresponds to the transition probability of the random walk with respect to which we need the stationary average. The transition probability *p* can belong to any random walk having uniform stationary distribution such that $q(\cdot |\cdot ) >0$ whenever $p(\cdot |\cdot ) >0$. One example is to use *p* as the transition probability of Metropolis–Hastings algorithm with target stationary distribution as uniform and *q* as the transition probability of a lazy version of simple random walk, i.e., with transition probability matrix $(\varvec{I}+\varvec{P}_{\scriptscriptstyle \text {SRW}})/2$. In comparison with basic Metropolis–Hastings sampling, such importance sampling avoids the API requests for probing the degree of all the neighboring nodes, instead requires only one such, viz., that of the sampled node. Note that the self-loops wherein the chain re-visits a node immediately are not wasted transitions, because it amounts to re-application of a map to the earlier iterate which is distinct from its single application.

The reinforcement learning scheme introduced above is the semi-Markov version of the scheme proposed in [20] and [21].

### Advantages

The RL-technique extends the use of regeneration, tours and super-node introduced in [4] to the average function ${\nu }(G)$. Even though the RL-technique is not non-asymptotically unbiased unlike the algorithm in [4], it has the following advantages:

1. It does not need to wait until burn-in time to collect samples;
2. Comparison with [4]: The super-node in [4] is a single node, an amalgamation of the node set $V_0$. But such a direction assumes that the contribution of all the edges inside the induced subgraph of $V_0$ to ${\nu }(G)$ completely known. It could have been avoided if we could make use of the techniques for partitioning state space of a Markov chain (called *lumpability* in [22]). The conditions stated in [22, Theorem 6.3.2] are not satisfied here and hence we can not invoke such techniques. But the RL-technique, without using the *lumpability* arguments, need not know the edge functions of the subgraph induced by $V_0$;
3. RL-technique along with the extension in “Extension of RL-technique to uniform stationary average case” section can further be extended to the directed graph case provided the graph is strongly connected. On the other hand, for estimators from other RW based sampling schemes, the estimator requires knowledge of the stationary distribution to unbias and thus to form the estimator. But in many cases including SRW on directed graphs, the stationary distribution does not have a closed form expression unlike in undirected case, and this poses a big challenge for design of simple random walk based estimators;
4. As explained before, the main advantage of RL-estimator is its ability to control the stability of sample paths and its position as a cross between deterministic and MCMC iteration. We will see more about this in the numerical section.

## Ratio with tours estimator (RT-estimator)

In this section, we use of the idea of regeneration and tours introduced in [4] to estimate the average function ${\nu }(G)$. However, since the tour estimator only gives an unbiased estimator for network sums namely $\sum _{i \in V} g(i)$, to find an estimate for ${\nu }(G)$ we use the same samples to get an estimate for ${ |V |}$. Let $I_n$ be the set of initial nodes recruited for forming the super-node [4] and let $S_n$ be the single combined node corresponding to $I_n$. We emphasize that while in RL-technique, the set of selected nodes $I_n$ stays intact, in the RT-estimator case, we shrink all these nodes in one super-node $S_n$. The estimator will compensate for network modification. With a sampling budget *B*, the RT-estimator is given by

9 $$\begin{aligned} \widehat{{\nu }}_{RT}( \mathcal {D}_{m(B)}(S_n)) = \dfrac{\sum \limits _{k=1}^{m(B)} \sum \limits _{t=1}^{\xi _k-1} \dfrac{f(X^{(k)}_t)}{d_{X^{(k)}_t}} + \dfrac{\sum _{i \in I_n} g(i)}{d_{S_n}}}{\sum \limits _{k=1}^{m(B)} \sum \limits _{t=1}^{\xi _k-1} \dfrac{1}{d_{X^{(k)}_t}} + \dfrac{n}{d_{S_n}}}, \end{aligned}$$

where *m*(*B*) is the number of tours until the budget *B*,

$$\begin{aligned} m(B) := \max \left \{k: \sum _{j=1}^k \xi _j \le B \right \}. \end{aligned}$$

The function $f(u) := g(u)$ if $u \notin I_n$, otherwise $f(u) =0$.

This estimator is very close to RDS sampling, explained in “Respondent-driven sampling technique (RDS-technique)” section, except that we miss $B-\sum _{k=1}^{m(B)} \xi _k$ samples for the estimation purpose and we use super-node to shorten tours. Namely, we can leverage all the advantages of super-node mentioned in [4, Section 2] and we claim that this would highly improve the performance. We show this via numerical simulations in the next section, and theoretical properties will be studied in the future.

Note that the formation of super-node is different from $V_0$ considered in the RL-technique, where the RW tours can start from any uniformly selected node inside $V_0$ and the tours end when it hit the set $V_0$. On the other hand, the super-node which is formed from *n* nodes in *V* is considered as a single node (removing all the edges in between the nodes in $S_n$) and this contracts the original graph *G*. Both the formulations have advantages of their own: Super-node and its estimator is easy to form and compute, but one needs to know all the edges between the nodes in $S_n$, i.e., the induced subgraph from $S_n$ should be known a priori. The set $V_0$ in RL-technique does not demand this.

## Numerical results

The algorithms RL-technique, RT-estimator, RDS, and MH-MCMC are compared in this section using simulations on three real-world networks. For the figures in this section, the *x*-axis represents the budget *B* which is the number of allowed samples, and is the same for all the techniques. We use the normalized root mean squared error (NRMSE) for comparison for a given *B* and is defined as

$$\begin{aligned} \text {NRMSE}:=\sqrt{\text {MSE}}/{\nu }(G),\quad \text {where } \text {MSE} = { \mathbb {E} \left[ {\left( \widehat{{\nu }}^{(n)}(G)-{\nu }(G) \right) }^2\right] }. \end{aligned}$$

### Datasets

We use the following datasets. All the datasets are publicly available.^3^

#### *Les Misérables network*

In Les Misérables network, nodes are the characters of the novel and edges are formed if two characters appear in the same chapter in the novel. The number of nodes is 77 and number of edges is 254. We have chosen this rather small network in order to compare the methods in terms of theoretical limiting variance (which is difficult to compute for large networks).

#### *Friendster network*

We consider a larger graph here, a connected subgraph of an online social network called Friendster with $64,\!600$ nodes and $1,\!246,\!479$ edges. The nodes in Friendster are individuals and edges indicate friendship.

#### Enron email network

The nodes in this network are the email ID’s of the employees in Enron and the edges are formed when two employees communicated through email. We take the largest connected component with 33, 696 nodes and 180, 811 edges.

### Choice of demonstrative functions

Recall that the network function average to be estimated $\nu (G) = \sum _{u \in V} g(u)/ |V|$. In the Les Misérables network, we consider four demonstrative functions: a) $g(v)=\mathbb {I}{\{d(v)>10 \}}$, b) $g(v)=\mathbb {I}{ \{d(v)<4 \}}$, c) $g(v)=d(v)$ where $\mathbb {I}{\{A\}}$ is the indicator function for set *A*, and d) for calculating ${\nu }(G)$ as the average clustering coefficient

10 $$\begin{aligned} C := \frac{1}{|V|} \sum _{v \in V} c(v),\quad \text {where } c(v) = {\left\{ \begin{array}{ll}t(v)/\left( {\begin{array}{c}d_v\\ 2\end{array}}\right) &{} \text {if } d(v) \ge 2 \\ 0 &{} \text {otherwise,} \end{array}\right. } \end{aligned}$$

with *t*(*v*) as the number of triangles that contain node *v*. Then *g*(*v*) is taken as *c*(*v*) itself. In the Friendster network, we consider the functions a) $g(v)=\mathbb {I}\{d(v)>50 \}$ and b) $g(v) = c(v)$ [see (10)] used to estimate the average clustering coefficient.

In case of the Enron network, we aim to estimate the proportion of number of elements in a community, assuming each node knows which community it associates with it. We look for community with index 1 with 6, 225 nodes, i.e., $g(v) = \mathbb {I}\{ \text {Comm}(v) = 1 \}$, with $\text {Comm}(v)$ denote the community node *v* part of. We expect such a function will have an impact on the performance of RDS and MH-MCMC. The communities are recovered using the algorithm described in [23]. We also consider $g(v)=d(v)$ for the Enron network.

### NRMSE comparison of RDS, MHMC, and RL-technique

For the RL-technique, we choose the initial set $V_0$ by uniformly sampling nodes assuming the size of $V_0$ is given a priori.

**Fig. 1:**
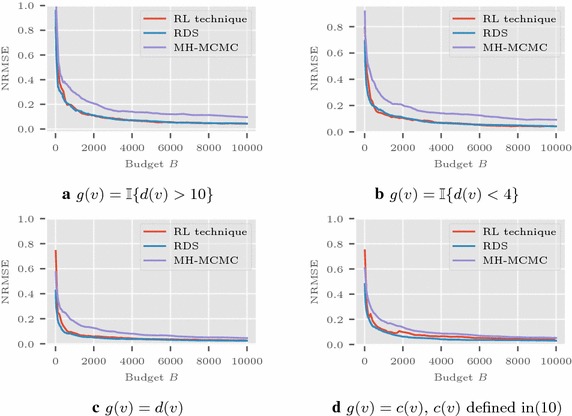
Les Misérables network: NRMSE comparisons

**Fig. 2:**
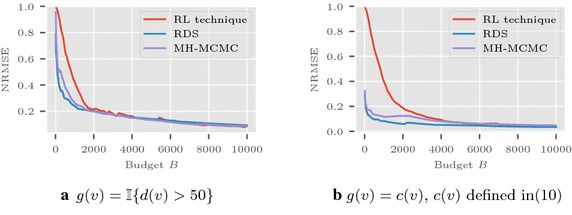
Friendster network: NRMSE comparisons

**Fig. 3:**
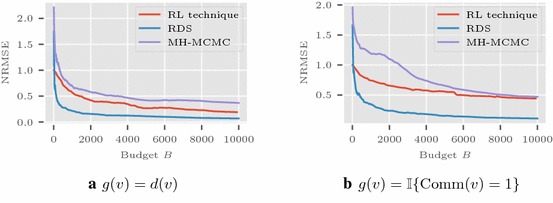
Enron email network: NRMSE comparisons

The average in MSE is calculated from multiple runs of the simulations. The simulations on Les Misérables network are shown in Fig. 1 with $a(n)=1/\lceil \frac{n}{10} \rceil$ and $|V_0|$ as 25. The plot in Fig. 2 shows the results for Friendster graph with $|V_0| = 1000$. Here the sequence *a*(*n*) is taken as $1/\lceil \frac{n}{25} \rceil$. Figure 3 presents the results in Enron network using $a(n) = 1/\lceil \frac{n}{20} \rceil$, and $V_0$ with 1000 nodes randomly selected.

Among the three techniques compared on these figures, RDS always works better. The MSE of RL-technique is comparable to RDS, and in some cases very close to it.

### Study of asymptotic variance of RDS, MHMC, and RL-technique

The mean squared error $\text {MSE} = \text {Var}[\widehat{{\nu }}^{(n)}(G)]+ {\Big ( \mathbb {E} [\widehat{{\nu }}^{(n)}(G)]-{\nu }(G) \Big )}^2$. Here we study the asymptotic variance $\sigma ^2_g$ [see (2)] of the estimators in terms of $n \times \text {MSE}$, since the bias $| \mathbb {E} [\widehat{{\nu }}^{(n)}(G)]-{\nu }(G) |\rightarrow 0$ as $n \rightarrow \infty$.

**Fig. 4:**
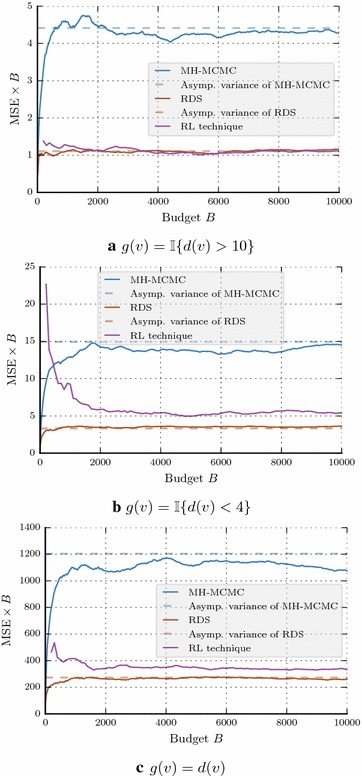
Les Misérables network: asymptotic variance comparisons (theoretical and estimated)

In order to show the asymptotic MSE expressions derived in Propositions 1 and 2, we plot the sample MSE as $\text {MSE} \times B$ in Figs. 4a–c. These figures correspond to the three different functions we have considered for Les Misérables network. It can be seen that asymptotic MSE expressions match well with the estimated ones.

### Stability of RL-technique

**Fig. 5:**
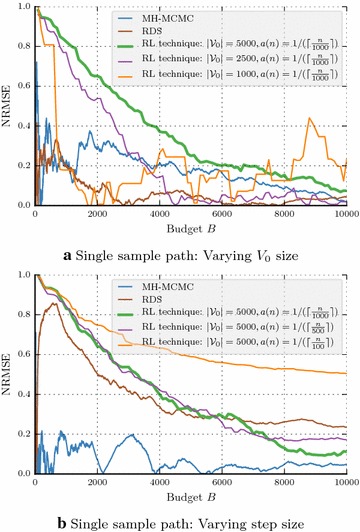
Friendster network: **a**, **b** Single sample path comparison with $g(v)=\mathbb {I}\{d(v)>50 \}$

Now we concentrate on *single* sample path properties of the algorithms. Hence, the numerator of NRMSE becomes absolute error. Figure 5a shows the effect of increasing initial set $V_0$ size while fixing step size *a*(*n*) and Fig. 5b shows the effect of changing *a*(*n*) when $V_0$ is fixed. In both the cases, the green curve of RL-technique shows much stability compared to the other techniques.

### Designing tips for the RL-technique

Some observations from the numerical experiments performed above are as follows:

1. With respect to the limiting variance, RDS always outperforms the other two methods tested. However, with a good choice of parameters the performance of RL is not far from that of RDS;
2. In the RL-technique, we find that the normalizing term $1/|V_0| \sum _j \mathcal {V}_n(j)$ converges much faster than the other two options, $\mathcal {V}_t(i_0)$ and $\min _i \mathcal {V}_t(i)$;
3. When the size of the super-node decreases, the RL-technique requires smaller step size *a*(*n*). For instance in case of Les Misérables network, if the initial set $V_0$ size is less than 10, RL-technique does not converge with $a(n)=1/(\lceil \frac{n}{50} \rceil +1)$ and requires $a(n)=1/(\lceil \frac{n}{5} \rceil )$;
4. If step size *a*(*n*) decreases or the super-node size increases, RL fluctuates less but with slower convergence. In general, RL has less fluctuations than MH-MCMC or RDS.

### NRMSE comparison of RDS and RT-estimator

Here, we compare RDS and RT estimators. The choice of RDS for comparison is motivated by the results shown in the previous section that it outperforms other sampling schemes considered in this paper so far, in terms of asymptotic variance and mean squared error. Moreover, RT-estimator can be regarded as a natural modification of RDS making use of the ideas of tours and super-node.

**Fig. 6:**
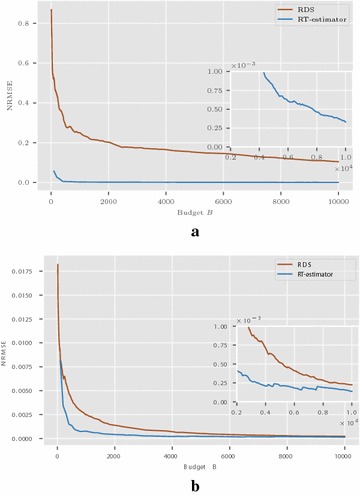
Friendster network: comparison between RDS and RT estimators

**Fig. 7:**
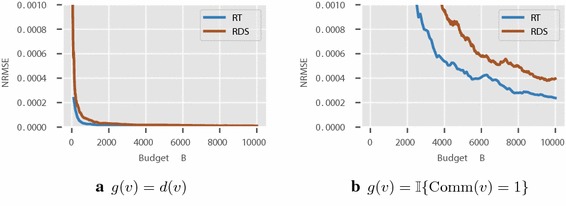
Enron email network: comparison between RDS and RT estimators

Figure 6 shows the results in Friendster network. It can be readily noticed that RT-estimator outperforms RDS. Figure 7 presents the results for Enron email network. In both the cases, RT-estimator performs the best and we see this as a consequence of the introduction of super-node to overcome slow mixing.

## Conclusions

We addressed a critical issue in the RW based sampling methods on graphs: the burn-in period. Our ideas are based on exploiting the tours (regenerations) and on the best use of the given seed nodes by making only short tours. These short tours or crawls, which start and return to the seed node set, are independent and can be implemented in a massively parallel way. The idea of regeneration allows us to construct estimators that are not marred by the burn-in requirement. We proposed two estimators based on this general idea. The first, the RL-estimator, uses reinforcement learning and stochastic approximation to build a stable estimator by observing random walks returning to the seed set. We then proposed the RT-estimator, which is a modification of the classical respondent-driven sampling, making use of the idea of short crawls and super-node. These two schemes have advantages of their own: the reinforcement learning scheme offers more control on the stability of the sample path with varying error performance, and the modified RDS scheme based on short crawls is simple and has superior performance compared to the classical RDS.

In the future, our aim is to study deeply the theoretical performance of our algorithms. We have also left open the selection process for the initial seed set or the super-node, and this also suggests an interesting research topic to explore in the future.

## Footnotes

1 https://dev.twitter.com/rest/public/rate-limits.

2 The underlying Markov chain of the RW requires to be irreducible in order to apply many results of the RWs and this is satisfied when the graph is connected. In case of a disconnected graph, taking at least one seed node from each of the components to form $V_0$ helps to achieve this.

3 See public repositories SNAP (https://snap.stanford.edu/data/) and KONECT (http://konect.uni-koblenz.de/).
